# Neural substrates of reward anticipation and outcome in schizophrenia: a meta-analysis of fMRI findings in the monetary incentive delay task

**DOI:** 10.1038/s41398-022-02201-8

**Published:** 2022-10-16

**Authors:** Jianguang Zeng, Jiangnan Yan, Hengyi Cao, Yueyue Su, Yuan Song, Ya Luo, Xun Yang

**Affiliations:** 1 School of Economics and Business Administration, Chongqing University, Chongqing, 400044 China; 2 Center for Psychiatric Neuroscience, Feinstein Institute for Medical Research, Hempstead, NY USA; 3 Division of Psychiatry Research, Zucker Hillside Hospital, Glen Oaks, NY USA; 4 School of Public Affairs, Chongqing University, Chongqing, 400044 China; 5 Department of Psychiatry, State Key Lab of Biotherapy, West China Hospital of Sichuan University, Chengdu, 610041 China

**Keywords:** Schizophrenia, Neuroscience

## Abstract

Dysfunction of the mesocorticolimbic dopaminergic reward system is a core feature of schizophrenia (SZ), yet its precise contributions to different stages of reward processing and their relevance to disease symptomology are not fully understood. We performed a coordinate-based meta-analysis, using the monetary incentive delay task, to identify which brain regions are implicated in different reward phases in functional magnetic resonance imaging in SZ. A total of 17 studies (368 SZ and 428 controls) were included in the reward anticipation, and 10 studies (229 SZ and 281 controls) were included in the reward outcome. Our meta-analysis revealed that during anticipation, patients showed hypoactivation in the striatum, anterior cingulate cortex, median cingulate cortex (MCC), amygdala, precentral gyrus, and superior temporal gyrus compared with controls. Striatum hypoactivation was negatively associated with negative symptoms and positively associated with the proportion of second-generation antipsychotic users (percentage of SGA users). During outcome, patients displayed hyperactivation in the striatum, insula, amygdala, hippocampus, parahippocampal gyrus, cerebellum, postcentral gyrus, and MCC, and hypoactivation in the dorsolateral prefrontal cortex (DLPFC) and medial prefrontal cortex (mPFC). Hypoactivity of mPFC during outcome was negatively associated with positive symptoms. Moderator analysis showed that the percentage of SGA users was a significant moderator of the association between symptom severity and brain activity in both the anticipation and outcome stages. Our findings identified the neural substrates for different reward phases in SZ and may help explain the neuropathological mechanisms underlying reward processing deficits in the disorder.

## Introduction

Schizophrenia (SZ) is one of the most severe neuropsychiatric disorders characterized by diverse symptoms including delusions, hallucinations, and thought disorders [1, 2]. Abnormal reinforcement learning and representations of reward value are present in SZ, and these impairments can manifest as deficits in reward decision making [3]. Accumulating evidence suggests that reward processing abnormalities in SZ patients may arise from dopaminergic dysfunction within the mesocorticolimbic circuit, including the dorsolateral prefrontal cortex (DLPFC), orbital prefrontal cortex (OFC), medial prefrontal cortex (mPFC), anterior cingulate cortex (ACC), ventral striatum (VS, including the nucleus accumbens), ventral pallidum, amygdala, hippocampus and thalamus [4–6]. Dysregulated dopaminergic modulation of reward processing is considered to be fundamental to the symptoms of SZ and is often reported to be an important predictor of poor functional outcome [7, 8].

Based on recent studies, reward processing includes two phases temporally, namely, the reward anticipation and reward outcome [9]. The monetary incentive delay (MID) task is the most widely used task to probe neural substrates of different reward processing stages in healthy individuals and those with mental disorders [9, 10]. In the MID task, subjects see a cue indicating that they will have an opportunity to obtain a certain amount of monetary reward, respond to a given target, and receive immediate feedback on whether they have successfully obtained the reward (see Supplementary Materials). The anticipation phase is defined by the introduction of a cue informing participants about an upcoming potential reward, and the outcome phase refers to the period when a reward is presented [11]. The investigation of reward processing in healthy adults revealed that anticipation of reward was associated with the activation of multiple regions including the striatum, ACC, anterior insula, and the central executive and default networks [11], while the OFC and mPFC were activated during the reward outcome phase [12]. This implies that the neural substrates of the two stages are likely to be associated with distinct patterns of activation and connectivity [12].

To date, present studies have examined and identified several likely neural substrates for the anticipation and outcome of incentives in SZ. However, due to the heterogeneity of reward paradigms and the bias introduced by including region of interest (ROI) analysis, the existing results are still inconsistent. During reward anticipation, although several studies revealed reduced VS activity in SZ [13–16], other studies reported reduced activations in the posterior cingulate cortex and temporal regions [17]. Compared with healthy controls (HC), activation in the VS in patients was found to be either reduced or not significantly changed (Supplementary Table 1). Reward anticipation abnormalities have been implicated in the pathophysiology of negative symptoms, such as anhedonia and avolition in SZ patients [16, 18]. However, there is also evidence suggesting correlations between anticipation dysfunction and the severity of positive symptoms [14, 19]. In terms of reward outcome, the role of striatum is relatively uncertain. Some behavioral and neuroimaging data have shown intact responses during the outcome phase [20, 21], while other data have shown either hyperactivity or hypoactivity in the striatum during monetary receipt [22–24]. Most studies report that outcome-related neural response in SZ patients is associated with both positive symptoms [25, 26] and negative symptoms [14]. Such inconsistencies may be attributed to the small sample size, sample heterogeneity, and differences in paradigm design among studies.

Currently, an updated quantitative meta-analysis method called seed-based mapping (SDM) has emerged as a useful approach to identify spatially consistent brain changes reported in the literature through the use of the coordinate information reported in each study. Few meta-analyses thus far have focused on dissociated neural responses during reward anticipation and outcome in SZ, although an increasing number of functional magnetic resonance imaging (fMRI) studies have reported potential neural substrates of reward processing. Radua and his colleagues used meta-analysis to reveal alterations in VS activity during reward anticipation, feedback, and prediction error [16]. However, VS activation in this meta-analysis was based on ROI approach, which would be affected by the different VS definitions across the included studies. Furthermore, both individuals with SZ and those at high risk for psychosis were included in the study, complicating the reported results. Another meta-analysis performed a whole-brain meta-analysis but only focused on the anticipation of reward tasks [27], while the neural substrates underlying different reward processing phases remain unclear.

Here, we performed an in-depth meta-analysis to elucidate the neurobiological basis underlying different stages of reward processing between SZ and HC. To overcome the limitations of previous meta-analysis work, we only included fMRI studies that performed a whole-brain analysis of patients as they completed the MID paradigm and analyzed reward anticipation and reward outcome independently. We also explored whether brain responses during different reward processes were associated with symptom severity using meta-regression analysis. We expected that reward anticipation and reward outcome would recruit different brain regions in SZ patients, and that the abnormal neural activations during different stages of reward processing would be closely related to the severity of symptoms.

## Materials and methods

### Study search and selection

**Fig. 1:**
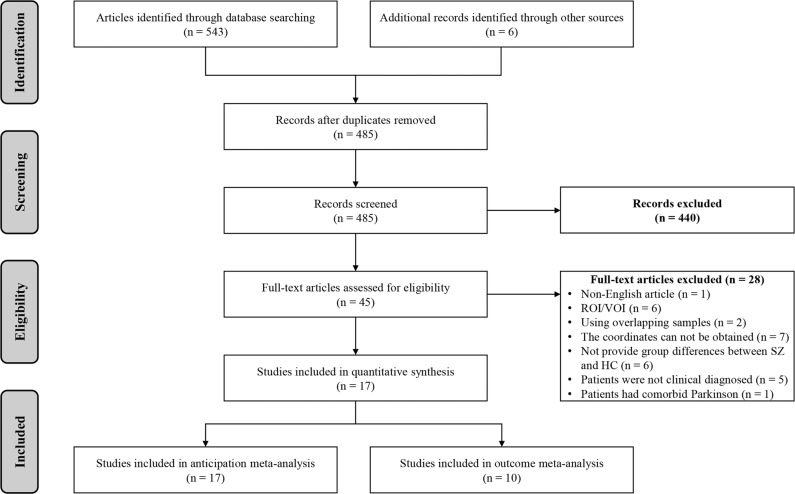
Preferred reporting items for systematic reviews and meta-analyses (PRISMA) flow diagram. Of 548 articles initially identified, a total of 17 studies were enrolled for the reward anticipation meta-analysis, and 10 studies were enrolled for the final reward outcome meta-analysis. MID monetary incentive delay, fMRI functional magnetic resonance imaging, ROI regions of interest, VOI volume of interest.

Following recommended guidelines in the Preferred Reporting Items for Systematic Reviews and Meta-Analyses statement [28], two authors independently searched the PubMed, Web of Science, and ScienceDirect databases for relevant articles from January 2000 to May 2021, using the following terms: (1) “schizophrenia” OR “schizophrenic” OR “schizoaffective” OR “psychoses” OR “psychosis” OR “psychotic” OR “first episode psychosis” OR “FEP”, (2) “functional magnetic resonance imaging” OR “fMRI” OR “neuroimaging”, and (3) “monetary incentive delay task” OR “MID”. We also manually examined the reference lists of the selected articles and relevant review articles to include more relevant studies. For studies without available coordinates at the whole-brain level, we asked the authors whether they could provide such information. Details of the literature search and selection were reported in Fig. 1.

Studies were eligible if they met the following criteria: (1) articles that included patients (aged >18 years) diagnosed with SZ, schizoaffective disorder, or another psychosis spectrum disorder based on the Diagnostic and Statistical Manual of Mental Disorders (DSM) or International Statistical Classification of Diseases and Related Health Problems (ICD) diagnostic criteria; (2) articles that investigated brain functional activity at the whole-brain level between adult SZ patients and HC; (3) articles that used a standardized MID task or modified MID task; (4) articles that examined neuronal activity related to the MID task using fMRI; (5) articles that identified foci of task-related neural changes in anticipation phase and/or outcome phase; and 6) articles that reported significant results as 3­D coordinates in either the Talairach Atlas (Tal) or Montreal Neurological Institute (MNI) space.

The exclusion criteria were as follows: (1) case reports, book chapters, reviews, or meta-analyses; (2) non-English articles; (3) studies that included only ROI or volume of interest (VOI) findings; and (4) studies in which the coordinates were not available in the article or after contacting the authors. Six fMRI studies did not provide group differences between SZ and HC and were thus not included in our meta-analysis [23, 29–33]. Of four studies using overlapping samples [14, 34–36], the one with more subjects [14] and the most recent study [35] were included. One study was excluded because the SZ patients had comorbid Parkinson’s disease [37].

### Data extraction

Data extraction was independently performed and checked by two authors. The following data were extracted: the sample sizes, the mean age, the percentage of males, the duration of illness, the severity of symptoms (Positive and Negative Syndrome Scale-Total (PANSS-T), PANSS-Positive (PANSS-P), PANSS-Negative (PANSS-N)), the proportion of SZ who had ever received first-generation antipsychotics (% (percentage) of FGA users)/ second-generation antipsychotics (% of SGA users), and methodological items.

### Meta-analysis of relevant studies

MID-related activation differences were analyzed using SDM (version 5.15, https://www.sdmproject.com), a novel voxel-based meta-analytic approach that uses the reported peak coordinates to recreate maps depicting the effect size of group differences in functional activations. Peak coordinates were recreated by first converting the peak *t* value to Hedges’ effect size and then applying a normalized Gaussian kernel to the voxels close to the peak. In addition to evaluating the probability of a peak, SDM can be used to recreate maps of the signed (i.e., positive and negative) functional activation or differences between patients and HC by using the reported peak coordinates, which makes SDM an optimal method for comparing patients and controls without biasing the results [38]. The statistical maps are created by calculating the corresponding statistics from the study maps and weighted by the squared root of the sample size of each study, amplifying the contributions of studies with larger sample sizes [38].

The SDM has more advantages than other methods, such as the arbitrary Lagrangian-Eulerian (ALE) method. First, instead of computing coordinates of increased and decreased activation separately, SDM can reconstruct both positive and negative differences in the same map [39]. Second, studies reporting no group differences can also be included. To date, SDM has been widely applied in previous meta-analyses of structural and functional MRI studies [40–44].

We closely followed the steps taken in the published literatures [45, 46]. In brief, peak coordinates of group differences and corresponding statistics were extracted from the included articles and then input into SDM software. Measurements (*z* scores and *p* values) were converted into *t* values in advance. Standard MNI maps of the activation differences were created using a Gaussian kernel, and the mean map was calculated representing the weighted mean functional differences during the MID task. Statistical significance was assessed by permutation testing. The default kernel size and statistical thresholds (full width at half maximum [FWHM] = 20 mm, *p* = 0.005, peak height threshold = 1, extent threshold = 10) were used to balance sensitivity and specificity [44, 46, 47].

In addition, to assess the robustness of the findings, complementary analyses were performed, including jackknife sensitivity analyses, subgroup analyses and meta-regression analyses. Based on the results of meta-regression, we also conducted moderation analyses using a standard model [48, 49] (see Supplementary Materials).

## Results

### MID-related brain activation differences between SZ and HC during reward anticipation

#### Included studies and sample characteristics

Seventeen studies with 368 SZ and 428 HC were included in the comparison of SZ and HC during reward anticipation [13–15, 17, 19, 22, 24–26, 35, 50–56] (Table 1 and Fig. 1). The mean age between SZ (32.10 years) and HC (32.36 years) was not significantly different (*t* = −0.524, *p* = 0.199). The percentage of males among SZ patients (75.00% male) and controls (70.01% male) was also not significantly different (*χ*^*2*^ = 2.382, *p* = 0.123).

**Table 1:** Demographic and clinical characteristics of the studies included in the meta-analysis.

Studies	Schizophrenia					Healthy controls		Methodology		
	Phase of illness	No. (male)	Mean age	Medication	Diagnosis criteria	No. (male)	Mean age	MRI scanner	SPM	Threshold
Anticipation stage										
Abler et al., [22]	Chronic SZ	12 (5)	36.70	SGA & FGA	DSM-IV	12 (7)	36.20	3 T	Y	Uncorrected, *p* < 0.005
Alves et al., [17]	FEP	10 (10)	22.70	SGA & FGA	DSM-IV	12 (12)	34.55	3 T	Y	Corrected, *p* < 0.05
Arrondo et al., [50]	Chronic SZ	22 (19)	32.73	SGA & FGA	DSM-IV	21 (17)	34.33	3 T	N	Corrected, *p* < 0.05
Esslinger et al., [19]	FEP	27 (20)	27.80	N	DSM-IV	27 (20)	27.10	3 T	Y	Corrected, *p* < 0.05
Gilleen et al., [51]	Chronic SZ	20 (20)	36.50	SGA & FGA	DSM-IV	12 (12)	30.70	3 T	Y	Corrected, *p* < 0.05
Juckel et al., [13]	Chronic SZ	10 (10)	26.80	N	DSM-IV & ICD-10	10 (10)	31.70	1.5 T	Y	Uncorrected, *p* < 0.001
Koch et al., [35]	Chronic SZ	44 (27)	34.20	SGA & FGA	DSM-IV & ICD-10	44 (35)	37.10	1.5 T	Y	Corrected, *p* < 0.05
Li et al., [24]	Chronic SZ	26 (15)	22.77	SGA	DSM-IV	26 (15)	24.58	3 T	Y	Corrected, *p* < 0.001
Mucci et al., [52]	Chronic SZ	28 (18)	33.10	SGA	DSM-IV	22 (10)	31.91	3 T	Y	Corrected, *p* < 0.05
Nielsen et al., [14]	FEP	31 (22)	25.90	N	ICD-10	31 (22)	25.70	3 T	N	Corrected, *p* < 0.05
Schlagenhauf et al., [56]	Chronic SZ	10 (9)	30.50	FGA	DSM-IV	10 (9)	31.80	1.5 T	Y	Corrected, *p* < 0.05
Schlagenhauf et al., [26]	Chronic SZ	15 (12)	30.10	N	DSM-IV	15 (12)	30.10	1.5 T	Y	Corrected, *p* < 0.05
Schwarz et al., [55]	Chronic SZ	27 (18)	32.40	SGA & FGA	DSM-IV	110 (54)	30.40	3 T	Y	Corrected, *p* < 0.05
Stepien et al., [53]	Chronic SZ	16 (14)	32.60	SGA	DSM-IV	23 (11)	29.50	3 T	Y	Corrected, *p* < 0.05
Subramaniam et al., [15]	Chronic SZ	37 (25)	45.14	SGA & FGA	DSM	20 (14)	43.72	3 T	Y	Uncorrected, *p* < 0.001
Walter et al., [54]	Chronic SZ	16 (8)	38.00	SGA	DSM-IV	16 (7)	33.00	3 T	Y	Uncorrected, *p* < 0.001
Waltz et al., [25]	Chronic SZ	17 (13)	37.80	SGA & FGA	DSM-IV	17 (12)	37.80	3 T	N	Corrected, *p* < 0.05
Outcome stage										
Abler et al., [22]	Chronic SZ	12 (5)	36.70	SGA & FGA	DSM-IV	12 (7)	36.2	3 T	Y	Uncorrected, *p* < 0.005
Gilleen et al., [51]	Chronic SZ	20 (20)	36.50	SGA & FGA	DSM-IV	12 (12)	30.7	3 T	Y	Corrected, *p* < 0.05
Li et al., [24]	Chronic SZ	26 (15)	22.77	SGA	DSM-IV	26 (15)	24.58	3 T	Y	Corrected, *p* < 0.001
Mucci et al., [52]	Chronic SZ	28 (18)	33.10	SGA	DSM-IV	22 (10)	31.91	3 T	Y	Corrected, *p* < 0.05
Nielsen et al., [14]	FEP	31 (22)	25.90	N	ICD-10	31 (22)	25.70	3 T	N	Corrected, *p* < 0.05
Schlagenhauf et al., [26]	Chronic SZ	15 (12)	30.10	N	DSM-IV	15 (12)	30.10	1.5 T	Y	Corrected, *p* < 0.05
Schwarz et al., [55]	Chronic SZ	27 (18)	32.40	SGA & FGA	DSM-IV	110 (54)	30.40	3 T	Y	Corrected, *p* < 0.05
Subramaniam et al., [15]	Chronic SZ	37 (25)	45.14	SGA & FGA	DSM	20 (14)	43.72	3 T	Y	Uncorrected, *p* < 0.001
Walter et al., [54]	Chronic SZ	16 (8)	38.00	SGA	DSM-IV	16 (7)	33.00	3 T	Y	Uncorrected, *p* < 0.001
Waltz et al., [25]	Chronic SZ	17 (13)	37.80	SGA & FGA	DSM-IV	17 (12)	37.80	3 T	N	Corrected, *p* < 0.05

*No*. number, *ICD-10* international statistical classification of diseases and related health problems, 10th Edition *DSM-IV* diagnostic and statistical manual of mental disorders, 4th Edition, *SZ* schizophrenia, *FEP* first episode psychosis, *HC* healthy controls, *SGA* second-generation antipsychotics, *FGA* first-generation antipsychotics, *Y* yes, *N* no.

#### Main meta-analysis

**Fig. 2:**
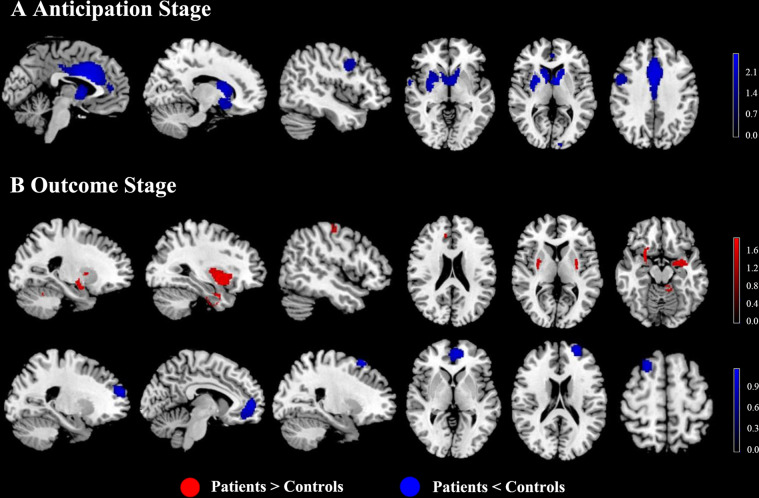
Task-evoked activation differences between SZ and HC during reward anticipation and reward outcome. **A** For the main analysis of the anticipation stage, SZ patients showed hypoactivation occurring in the bilateral striatum, ACC, MCC, amygdala, right precentral gyrus, and right STG, compared with HC. **B** For the main analysis of the outcome stage, patients showed hyperactivation in the striatum (with extension to the bilateral insula, amygdala, and hippocampus), left cerebellum, right parahippocampal gyrus, right postcentral gyrus, and right MCC, and hypoactivation in the mPFC and DLPFC compared with HC. Brain regions that showed significant differences during the outcome stage in SZ patients relative to HC. Red indicates regions that showed hyperactivation in SZ compared with HC and blue indicates regions that showed hypoactivity in patients relative to HC. The color scale represents probability values from statistical permutation testing (*z* values). SZ schizophrenia, HC healthy controls, ACC anterior cingulate cortex, MCC median cingulate cortex, STG superior temporal gyrus, mPFC medial prefrontal cortex, DLPFC dorsolateral prefrontal cortex.

In the pooled meta-analysis of reward anticipation, relative to HC, SZ exhibited lower activations in the striatum (with extension to the insula and amygdala), ACC, median cingulate cortex (MCC), right precentral gyrus and right superior temporal gyrus (STG) in response to monetary stimuli. No brain regions showed increased activation in SZ patients compared to HC (Table 2 and Fig. 2).

**Table 2:** Results of the meta-analysis for brain activation difference between SZ and HC during reward anticipation stage.

Brain regions	MNI	SDM value	*p* value	Number of voxels	Breakdown
	coordinates				
	*x*, *y*, *z*				
*P* < HC					
Bilateral striatum	−8,4,6	−2.662	~0	2956	Right striatum Right lenticular nucleus, putamen, BA 11, BA 25, BA 48 Right caudate nucleus Left caudate nucleus, BA 25 Right caudate nucleus, BA 11, BA 25 Right olfactory cortex, BA 11, BA 25, BA 48 Right amygdala, BA 34, BA 48 Left striatum Right inferior network, uncinate fasciculus, inferior fronto-occipital fasciculus Right gyrus rectus, BA 11, BA 25, BA 48 Right superior longitudinal fasciculus III Right insula, BA 48 Right median network, cingulum Left olfactory cortex, BA 25 Right hippocampus, BA 34
ACC & MCC	0,12,24	−2.620	0.000005186	1931	Left median cingulate / paracingulate gyri, BA 23, BA 24, BA 32 Right median cingulate / paracingulate gyri, BA 23, BA 24, BA 32 Left anterior cingulate / paracingulate gyri, BA 10, BA 24, BA 32 Left median network, cingulum Right anterior cingulate / paracingulate gyri, BA 24, BA 32 Right median network, cingulum Left superior frontal gyrus, medial, BA 8, BA 24, BA 32 Left supplementary motor area, BA 8, BA 24, BA 32 Right superior frontal gyrus, medial, BA 32 Right supplementary motor area, BA 32
Right precentral gyrus	50,4,36	−1.919	0.000526428	244	Right precentral gyrus, BA 4, BA 6, BA 44 Right middle frontal gyrus, BA 6, BA 9, BA 44 Right inferior frontal gyrus, opercular part, BA 44 Right postcentral gyrus, BA 4, BA 6
Right STG	62,0,−4	−1.693	0.001878560	30	Right superior temporal gyrus, BA 21, BA 38, BA 48 Right temporal pole, superior temporal gyrus, BA 21, BA 38, BA 48

Results were threshold at *p* = 0.005, peak height threshold of 1, extent threshold of 10.

*BA* Brodmann area, *P* patients, *HC* healthy controls, *ACC* anterior cingulate cortex, *MCC* median cingulate cortex, *STG* superior temporal gyrus, *SDM* seed-based d mapping, *MNI* Montreal Neurological Institute.

#### Sensitivity analysis

As illustrated in Supplementary Table 2, whole-brain jackknife sensitivity analysis confirmed that hypoactivation in the bilateral striatum, ACC and MCC maintained significance in all but one combination. The results in the right precentral gyrus and right STG maintained significance in all but two combinations.

#### Subgroup analyses

To control for any possible differences observed between studies, subgroup analyses were repeated several times to include only those studies that were clinically and methodologically homogenous. Therefore, we conducted subgroup analysis for those studies only including chronic SZ, for those including SZ patients diagnosed by DSM, for those including SZ patients receiving medication treatment, for those using a 3-T MRI scanner, for those using SPM software, and for those reporting coordinates corrected for comparisons. The subgroup analysis revealed that all of the aforementioned results were highly replicable, except for decreased activation in the right precentral gyrus and right STG (Supplementary Table 2).

#### Meta-regression analyses

**Fig. 3:**
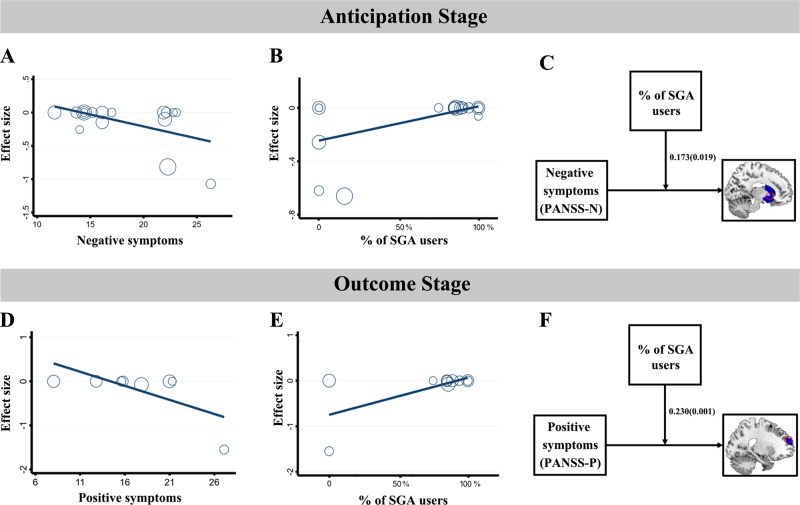
Correlations and moderation analyses between clinical symptoms and brain activity during reward anticipation and reward outcome. **A** Scatter plot showing a significant negative association between anticipation-evoked activity and negative symptom severity (PANSS-N) in the VS (MNI coordinates: *x* = 16, *y* = 14, *z* = −6, *r* = −0.507, *p* = 0.038). **B** Scatter plot showing a significant positive association between anticipation-evoked activity and the % (percentage) of SGA users (the proportion of SZ who had ever received SGA) in the VS (MNI coordinates: *x* = 16, *y* = 2, *z* = 0, *r* = 0.533, *p* = 0.019). **C** Conceptual diagram of the moderating effect of the % of SGA users on the relationship between negative symptoms and striatum hypoactivation during reward anticipation. **D** Scatter plot showing a significant negative association between outcome-evoked activity and the positive symptom severity (PANSS-P) in the mPFC (MNI coordinates: *x* = 0, *y* = 46, *z* = −10, *r* = −0.681, *p* = 0.043). **E** Scatter plot showing a significant positive association between outcome-evoked activity and the % of SGA users in the mPFC (MNI coordinates: *x* = 0, *y* = 46, *z* = −10, *r* = 0.656, *p* = 0.028). **F** Conceptual diagram of the moderating effect of % of SGA users on the relationship between positive symptoms and mPFC hypoactivation during reward outcome. SZ schizophrenia, HC healthy controls, SGA second-generation antipsychotic, mPFC medial prefrontal cortex.

We next tested correlations between anticipation-evoked activations and demographic and clinical variables, including the mean age, the percentage of males, the duration of illness, the symptom severity and the medication variables. There were no significant correlations between brain MID-related activations and mean age (available in 17 studies), between brain MID-related activations and percentage of males (available in 17 studies), between brain MID-related activations and duration of illness (available in 12 studies), between brain MID-related activations and PANSS-P (available in 14 studies), and between brain MID-related activations and the % of FGA users (available in 17 studies) during reward anticipation. Meta-regression analyses revealed that the severity of negative symptoms (available in 15 studies) was negatively associated with anticipation-evoked hypoactivation in the VS (MNI coordinates: *x* = 16, *y* = 14, *z* = −6, *r* = −0.507, *p* = 0.038). In addition, the % of SGA users (available in 17 studies) was positively related to the VS activation (MNI coordinates: *x* = 16, y = 2, z = 0, *r* = 0.533, *p* = 0.019) (Fig. 3).

#### Moderation analyses

Since the negative symptom severity was significantly negatively related to striatum hypoactivation (*r* = −0.507, *p* = 0.038), we further tested whether the symptom-brain association was dependent on a third variable (including the age, the sex and the medication) through the moderation analysis. Our moderation analysis revealed that the interactions of brain × age and brain × sex were not statistically significant. In the test of the moderation effect of medication, the interaction of the % of SGA users and negative symptoms significantly improved model fit, suggesting a moderating effect of the % of SGA users on the relationship between negative symptom severity and VS activity (*R*^*2*^ change = 0.263, *B* = 0.173, *p* = 0.019). That is, for individuals in the lower SGA group, a higher PANSS-N score was associated with more decreased striatum activation, while for individuals in the higher SGA group, negative symptoms presented a null association with striatum activity (Fig. 3 and Supplementary Table 3).

### MID-related brain activation differences between SZ and HC during reward outcome

#### Included studies and sample characteristics

Regarding the reward outcome stage, a total of ten studies comprising 229 SZ and 281 HC met the meta-analysis inclusion criteria [14, 15, 22, 24–26, 51, 52, 54, 55] (Table 1 and Fig. 1). The mean age between SZ (33.84 years) and HC (32.41 years) was not significantly different (*t* = −0.539, *p* = 0.596). There was no significant difference (*χ*^*2*^ = 0.601, *p* = 0.438) in the percentage of males between SZ patients (67.60% male) and controls (64.59% male).

#### Main meta-analysis

Compared with HC, SZ showed higher activations in the bilateral striatum (with extension to the bilateral insula, amygdala, and hippocampus), left cerebellum, right parahippocampal gyrus, right postcentral gyrus, and right MCC during the outcome stage. Significant lower activations in SZ patients were detected in the mPFC and bilateral DLPFC compared to HC (Table 3 and Fig. 2).

**Table 3:** Results of the meta-analysis for brain activation difference between SZ and HC during reward outcome stage.

Brain regions	MNI	SDM value	*p* value	Number of voxels	Breakdown
	coordinates				
	*x*, *y*, *z*				
*P* > HC					
Right striatum	28,10,−14	1.829	0.000118673	590	Right lenticular nucleus, putamen, BA 48 Right striatum Right amygdala, BA 34, BA 48 Right olfactory cortex, BA 11, BA 48 Right inferior network, inferior fronto-occipital fasciculus Right parahippocampal gyrus, BA 34, BA 48 Right insula, BA 48 Right superior frontal gyrus, orbital part, BA 11 Right inferior frontal gyrus, orbital part, BA 11, BA 48 Right lenticular nucleus, putamen, BA 47 Right temporal pole, superior temporal gyrus, BA 34 Right hippocampus, BA 34
Left striatum	−22,0,2	1.696	0.000289023	439	Left amygdala, BA 20, BA 28, BA 34 Left striatum Left lenticular nucleus, putamen, BA 48 Left insula, BA 48 Left pons Left inferior network, uncinate fasciculus, inferior longitudinal fasciculus, inferior fronto-occipital fasciculus Left hippocampus, BA 34 Left superior temporal gyrus, BA 48
Left cerebellum	−8,−36,−18	1.443	0.001243770	194	Left cerebellum, hemispheric lobule IV / V, BA 19, BA 30,, BA 37 Left cerebellum, hemispheric lobule VI, BA 19, BA 37 Left cerebellum, hemispheric lobule III, BA 30 Middle cerebellar peduncles Left cerebellum, hemispheric lobule VI, BA 18
Right parahippocampal gyrus	30,−4,−26	1.676	0.000314832	176	Right fusiform gyrus, BA 20, BA 36 Right inferior network, inferior longitudinal fasciculus, uncinate fasciculus Right parahippocampal gyrus, BA 20, BA 28, BA 35, BA 36 Right median network, cingulum Right amygdala, BA 28, BA 36 Right hippocampus, BA 36
Right postcentral gyrus	52,−18,56	1.443	0.00120765	31	Right postcentral gyrus, BA 4, BA 6 Right precentral gyrus, BA 4, BA 6 Right middle frontal gyrus, BA 6
Right MCC	8,34,30	1.438	0.001398563	20	Right median cingulate / paracingulate gyri, BA 32
*P* < HC					
mPFC	−10,52,6	−1.129	0.000010312	817	Left anterior cingulate / paracingulate gyri, BA 10, BA 11, BA 32 Left superior frontal gyrus, medial, BA 10, BA 32 Left superior frontal gyrus, medial orbital, BA 10, BA 11 Right superior frontal gyrus, medial orbital, BA 10, BA 11 Left gyrus rectus, BA 11 Left median network, cingulum Right superior frontal gyrus, medial, BA 10 Right gyrus rectus, BA 11 Right anterior cingulate / paracingulate gyri, BA 10, BA 11
Left DLPFC	−24,56,20	−1.033	0.000051618	442	Left middle frontal gyrus, BA 9, BA 10, BA 46 Left superior frontal gyrus, dorsolateral, BA 9, BA 10, BA 46
Right DLPFC	22,20,62	−1.036	0.000025809	143	Right superior frontal gyrus, dorsolateral, BA 8

Results were threshold at *p* = 0.005, peak height threshold of 1, extent threshold of 10.

*BA* Brodmann area; *P* patients; *HC* healthy controls, *MCC* median cingulate cortex, *mPFC* medial prefrontal cortex, *DLPFC* dorsolateral prefrontal cortex, *SDM* seed-based d mapping, *MNI* Montreal Neurological Institute.

#### Sensitivity analysis

The jackknife sensitivity analysis revealed that the findings in the left striatum, right parahippocampal gyrus, and right postcentral gyrus were consistent in all combinations of studies. The increased activations in the right striatum left cerebellum and right MCC, as well as reduced activations in the mPFC and DLPFC, remained significant except for one combination (Supplementary Table 4).

#### Subgroup analyses

Similarly, we conducted subgroup analyses for those studies only including chronic SZ, for those including SZ patients diagnosed by DSM, for those including SZ patients receiving medication treatment, for those using a 3-T MRI scanner, for those using SPM software, and for those reporting coordinates corrected for comparisons. The results remained largely unchanged in all of the subgroup analyses (Supplementary Table 4).

#### Meta-regression analyses

For reward outcome, the effect of duration of illness could not be examined due to insufficient data (available in six studies). No significant correlations were found between outcome-evoked activations and several variables (the percentage of males (available in 10 studies), the % of FGA users (available in 10 studies), and the PANSS-N scores (available in eight studies)). Hypoactivation in left mPFC was found to be negatively associated with the PANSS-P scores (MNI coordinates: *x* = 0, *y* = 46, *z* = −10, *r* = −0.681, *p* = 0.043; available in eight studies). Moreover, the % of SGA users was found to be positively related to the left mPFC activation (MNI coordinates: *x* = 0, *y* = 46, *z* = −10, *r* = 0.656, *p* = 0.028; available in 10 studies) (Fig. 3).

#### Moderation analyses

During reward outcome, a similar pattern of findings emerged for the moderating effect of % of SGA users. In detail, the interaction of SGA and positive symptoms was a significant predictor of mPFC activity (*R*^*2*^ change = 0.457, *B* = 0.230, *p* = 0.001). A higher PANSS-P was associated with more decreased mPFC activation among participants in the lower SGA group, whereas among participants in the higher SGA group, PANSS-P scores had a null association with mPFC activity (Fig. 3 and Supplementary Table 5).

## Discussion

Our whole-brain meta-analysis of fMRI studies stressed the importance of examining the temporal phases (i.e., anticipation and outcome) of reward processing separately, as we showed dissociable neural substrates during reward anticipation and receipt. During reward anticipation, individuals with SZ showed a reduced response to reward in the mesocorticolimbic circuitry involving the striatum, insula, ACC, MCC, amygdala, right precentral gyrus, and right STG. In contrast, during reward outcome, individuals with SZ showed increased activation in the striatal-limbic circuitry involving the bilateral striatum, insula, amygdala, hippocampus, right parahippocampal gyrus, left cerebellum, right postcentral gyrus, right MCC, and decreased activation in the mPFC and DLPFC when processing incentive feedback. In addition, anticipation-evoked activation reductions in the VS were negatively correlated with negative symptoms of SZ, whereas outcome-evoked activation reductions in the mPFC were negatively correlated with positive symptoms of SZ. The relationship between symptom severity and brain activity was moderated by the % of SGA users. This meta-analysis provided evidence that different brain regions in SZ patients are implicated in reward anticipation and reward outcome during the MID task.

### Anticipation-evoked brain responses in SZ

During anticipation, we found that SZ patients exhibited reduced activation in the mesocorticolimbic reward system in response to monetary incentives. Dopamine neurons in the midbrain project widely to the cortex and subcortical structures, including the VS, dorsal striatum, amygdala, thalamus, and hippocampus. These dopaminergic pathways play an important role in the modulation of motivational processing and decision-making, and changes in dopamine metabolism are thus considered as the central basis for the impairment of “wanting” and “learning” -related physiology in SZ. “Wanting” refers to the motivational processing of the incentive salience attributed to the reward and is mediated by larger systems that encompass mesocorticolimbic dopaminergic transmission [57]. Generally, increases in dopaminergic transmission are associated with increases in motivated behavior, whereas disruption of dopaminergic functioning, through focal lesions or pharmacologically induced receptor antagonism/depletion, reduces motivated behavior [58]. VS response during reward anticipation has been previously shown to be attenuated in drug-naive patients, patients with chronic SZ, and individuals at high risk for psychosis [13, 19, 59]. Moreover, the degree of reduced reward anticipation is linked to symptom severity [36]. Thus, our findings of reduced striatal activity in SZ when anticipating a monetary reward, likely reflect a blunted attribution of motivational salience to monetary stimuli.

Reward anticipation is also associated with the deactivations of the ACC and insula region, as proposed by the aberrant salience hypothesis [60, 61]. Aberrant salience refers to an abnormally reduced response to a reward or related stimuli but a heightened response to neutral or irrelevant stimuli. The salience network (SN) which mainly comprises the ACC and insula, is thought to cause such aberrant salience attribution [62]. The ACC has extensive connections with a set of other limbic and related areas including the amygdala, OFC, and STG, and is involved in reward-related processing by encoding reward outcomes and determining the effort required to obtain rewards [63]. The insular cortex has emerged in the last few years as a key region in SZ research and is considered to be a crucial relay center of interoceptive signals that integrates with exteroceptive awareness [64]. A previous major depressive disorder (MDD) study showed that the functional activity and functional connectivity (FC) of the insula were important indicators of electroconvulsive therapy [65]. Several SZ studies have reported the reduced gray matter and/or functional activity and FC within the insula–ACC SN [66, 67]. Palaniyappan and colleagues have proposed that dysfunction of the insula–ACC SN is linked to the psychotic symptoms of SZ by inappropriately allocating salience to irrelevant internal or external stimuli [68]. Reduced FC within brain regions involving the ACC and insula was proven to be associated with heightened affective and anxiety symptoms and an increased risk of developing psychiatric disorders [69, 70]. Incentive valence, behavioral relevance, or expectancy violation would determine the processing the stimulus salience of momentary reward and lead to a change in the brain state [68, 71]. Therefore, abnormalities in the mesolimbic system and the cortical SN detected when individuals are performing the MID task may help explain abnormal reinforcer processing and symptoms, which appears to be a prominent characteristic of the pathophysiology of SZ.

Blunted activations in the right STG and precentral gyrus of SZ patients in response to monetary stimuli were also found during anticipation. Decreased gray matter volume and changed functional activation and FC in the STG have been robustly implicated in the neurophysiology of SZ [72–74]. Pertinently, the STG plays a key role in language perception, which is consistent with previous reports linking this region with auditory hallucinations in SZ [75]. However, the temporal area is sensitive to socially relevant information and may also be linked to incentive salience processing [76]. The precentral gyrus has also been associated with behavioral responses to motivationally significant events [77]. Abnormally reduced reward-related activity in the precentral gyrus and STG in SZ would imply a reduction in salience to rewarding events, as well as motivated goal-directed behavior by associations with reinforcing events.

### Outcome-evoked responses in SZ

In contrast, at the reward outcome stage, SZ showed elevated activations in the striatal-limbic circuitry, including the bilateral VS, amygdala, insula, hippocampus, right parahippocampal gyrus, left cerebellum, right postcentral gyrus, and right MCC, and diminished activations in the mPFC and DLPFC. Impaired neural processing during reward outcome may be independent from motivational components given that hedonic impact was found to be independent from anticipation effects. Our meta-analysis revealed that SZ showed stronger activation in the VS during the receipt of a reward, perhaps indicating an elevated reactivity to rewarding outcomes. Consistent with our findings, past research reported that striatal activation is associated with reward outcome and that SZ patients reveal higher striatum signals during the outcome phase [54]. Furthermore, animal studies supported the involvement of the VS in the experience of pleasure and hedonic perception of rewards [78]. As part of the limbic structures, the VS, amygdala, and insula are thought to play a significant role in guiding behavior and facilitating learning. A growing body of evidence suggests that the amygdala is critical for feedback-guided learning behavior, and VS reflects the encoding of expected value of outcome and action selection for the obtainment of rewards [79, 80], whereas the parahippocampal gyrus is related to prediction errors [81, 82]. A similar pattern of activation within limbic regions has been reported during the receipt of a reward [22, 25, 54]. Regarding the preferential involvement of the limbic-striatal areas in hedonic processes, the findings of over-responsiveness to rewarding outcome may reflect the presumed motivational significance of hedonic experience underlying the reward-seeking behavior.

Outcome-related increased activity was also present in the cerebellum in SZ patients. Evidence indicates that the cerebellum plays a role in higher cortical functions, such as emotional processing and social cognition [83, 84]. It has been proposed that the cerebellum encodes error signals and participates in feedback-based learning. Recent findings of altered error processing in patients with cerebellar lesions confirmed the hypothesis that feedback processing might be affected by cerebellar damage [85, 86]. Notably, cerebellar dysfunction and hyperconnectivity patterns in the cerebello-thalamo-cortical circuit have been consistently observed in SZ [87–89]. Our finding of exaggerated cerebellar activation in the reward reception phase appears to reflect the importance of cerebellum in controlling the reward process in psychosis.

Reduced activation in response to reward was observed in the DLPFC and mPFC during the outcome. Pertinently, the prefrontal cortex is a heterogeneous area that is critical to reward-based decision-making. Studies have demonstrated that the DLPFC is implicated in higher-order cognitive control, especially for reward values and effort calculations [90, 91], whereas the mPFC is a key node for emotion-related reward processes and value-based decision-making through interactions with the VS and amygdala [92, 93]. Furthermore, reduced FC in medial prefrontal-striatal network is related to disrupted cognitive control and reward processing [94]. Promoting local, long-range, and dynamic connectivity within the frontal areas could effectively improve cognitive function [95]. Because the PFC exerts top-down control over striatal dopamine-induced activity and drives synchrony between specific corticolimbic circuit regions [96], we speculated that reduced cortical excitability in the prefrontal region might trigger elevated striatal and limbic responses.

### Correlations between clinical symptoms and brain activity at different reward stages

During reward anticipation, the meta-regression analysis revealed a significant negative correlation between VS hypoactivation and the severity of negative symptoms. In line with this, a strong association of reduced VS activation during reward anticipation with negative symptoms was observed in previous studies [13, 16]. As the activity of this region mediates incentive motivation or wanting of reward, this result may suggest that impaired striatal activity is involved in the pathophysiology of motivational deficits in SZ patients. A similar result was observed in our meta-analysis in the association with current antipsychotic drug use. Specifically, we found that VS hypoactivation was positively associated with the % of SGA users. Consistent with this finding, Juckel and colleagues reported an improvement in reduced VS activity in patients taking SGA but not in those taking FGA or those who were unmedicated [97]. Since SGA has less blockade of striatal D_2_ receptors, it may enhance the treatment of negative symptoms and maintain motivation to reach potential rewards owing to less blockade of striatal D_2_ receptors [98]. In this regard, the linkages between blunted striatal anticipating function and negative symptoms would help to explain the different facets of reward processing in the correlation of behavioral disturbances.

During reward outcome, our meta-regression analysis further revealed that reward-related hypoactivity in the mPFC was negatively associated with positive symptoms. Dysfunction in the mPFC may result in hallucinations and delusions [26]. Several postmortem and fMRI studies have provided evidence for abnormal anatomical and FC of the mPFC, which is implicated in psychiatric symptoms and impaired cognitive function in SZ and MDD patients [99, 100]. For example, previous studies found that hyperconnectivity between the mPFC and default mode network was correlated with more serious positive symptoms in SZ patients [101]. Along similar lines, an association between disrupted error feedback in the mPFC and delusion severity was observed [102]. Our study also found that mPFC activation was positively associated with the % of SGA users during the outcome phase. As mentioned above, SGA administration could improve the dysfunction of the PFC and positive symptoms. Our meta-regression results suggested that the neural processing of reward outcomes in the mPFC may be related to the pathophysiology of positive symptoms in SZ patients. Notably, although the regression analysis results during reward outcome are statistically significant, they are preliminary and require future research to obtain a better understanding of their effects.

Our exploratory moderation analyses revealed that SGA use was a significant moderator of the symptom-brain relationship during reward anticipation and outcome: results were negative in patients taking fewer SGAs and null in patients taking more SGAs. In patients in the group taking more SGA, negative symptoms presented a null association with brain activity. It is well documented that SGA is presumed to act as a treatment for negative symptoms [103] and multiple neuroprotective effects on the brain [104]. It is likely that SGA affect symptoms and the brain simultaneously, and thereby reduces and weakens the link between symptoms and brain activity in patients taking more SGA; On the other hand, in patients taking fewer SGA, the symptoms were inversely related to brain activity. In accordance, previous reports show that the more serious negative symptoms are, the stronger the reduction in striatal activation under the condition that patients took fewer SGA [13, 35]. Moreover, the striatal activation reduction was inversely correlated with the severity of negative symptoms in patients being not treated with SGA [98]. A recent European Psychiatric Association guidance paper argued SGA (i.e., amisulpride) has certain potential in the treatment of negative symptoms and suggested that a switch to SGA should be considered for patients who are treated with FGA [105]. additionally, a randomized controlled trial revealed that SGA showed statistically significant effects on negative symptoms [106]. In the present study, we present preliminary evidence for a moderating role of SGA in the relationship between clinical symptoms and brain activity. The symptom-brain relationship is complex, and further studies of how this relationship changes as modulation of antipsychotic treatment need to be validated in controlled clinical trials.

### The heterogeneity in the subgroup analyses of anticipation stage

Notably, the results showed that the brain activity in the right STG did not survive in the subgroup analyses for studies including chronic SZ patients and studies including SZ patients receiving medication treatment. The medial-temporal lobe, including the STG, is probably the most extensively investigated brain structure in SZ. STG is believed to be a major anatomical substrate for auditory hallucinations and thought disorders in SZ [107]. Although STG abnormalities have been well demonstrated in SZ, some studies have reported negative results for STG abnormalities [36, 51, 53]. A meta-analysis of voxel-based morphometry in SZ reported that 6 of 15 studies showed no significant brain volume difference in the STG when compared with controls [108]. Furthermore, an attenuated response in the STG during the anticipation of monetary incentives has been found in medication-free SZ patients but not in medicated patients [14, 36]. It is well known that SZ is a chronic psychiatric disorder that can be effectively controlled but likely requires lifelong treatment. Previous neuroimaging studies of SZ examined chronically ill patients, for whom findings are potentially influenced by disease course and medication. Exposure to antipsychotic drugs may have an effect on brain structure and function [109–111]. For example, increased cortical thickness and increased anticipation-related brain activity in the STG over treatment time have been observed, which is associated with symptomatic improvement [36, 112]. Importantly, it has been suggested that the improvement of positive symptoms was significantly associated with the normalization of reward-related activation [36]. SZ patients who show a long illness duration may also experience a neurotoxic effect on their brain structure and function [113, 114]. Furthermore, the STG can be cytoarchitectonically and functionally divided into several subdivisions [115]. The complexity and heterogeneity of the STG may account for the inconsistency.

In addition, our current subgroup analysis of studies that applied a 3-T MRI scanner also found that STG activity showed some heterogeneity. Different studies used different MRI scanners with different MRI field strengths, which could introduce potential bias. One possible explanation for this is that in a high-strength field, echo planar imaging results in a higher signal-to-noise ratio but also increases susceptibility artifacts [116]. In particular, it is influenced in regions with susceptibility artifacts, especially for imaging the temporal lobes [117]. Future studies should investigate the influence of different magnetic strengths on imaging presentation, and meta-analyses with homogeneous magnetic field strength are needed to confirm this finding.

### Clinical implications

The biological markers of different stages of reward processing may help elucidate the complex and multifaceted symptoms as well as neurobehavioral disruptions observed in SZ patients. Dysfunction in reward processing is regarded in the DSM-5 as a key factor in the anhedonic symptoms of SZ [118]. Previous studies found that SZ patients have impaired motivation to pursue rewards and reduced activation in the reward pathway during the presentation of reward stimuli, although pleasure in consuming rewards is largely intact [7]. Deficits in any reward component, including reward valuation, reward expectancy, and action selection, may preclude an individual from engaging in goal-directed actions for rewards, regardless of whether the reward is perceived as pleasurable once obtained. In other words, the construct of anhedonia that reflects deficits in hedonic capacity is closely linked to the constructs of reward anticipation, valuation, and motivation. In our meta-analysis, we found that anticipation and outcome stages of reward may recruit distinct activation patterns, and these patterns are correlated with different clinical symptoms. As SZ is linked to changes in reward processing, probing distinct neural processes of the reward system may help improve the present understanding of the role of different aspects of reward system in the pathogenesis of SZ.

Distinguishing the reward anticipation and outcome phases may also reveal a biomarker that can be used to predict treatment outcomes. A recent study with healthy human volunteers provided pharmacological evidence that the effort to obtain a reward and the related facial reactions during reward anticipation are modulated by the administration of dopaminergic antagonists [119]. Another study observed that reward anticipation activity, but not reward outcome activity, was significantly associated with an antidepressant response in individuals with major depression disorder [120]. The authors also observed that the frontostriatal connectivity during reward anticipation was significantly correlated with a reduction in depressive symptoms [120]. Consistent with these findings, SGA influences VS activation during anticipation and mPFC activation during the delivery of a monetary reward in SZ. Interestingly, our findings showed dissociated neurobiological mechanisms in different aspects of reward processing, and have the potential to clarify the complex brain-behavior relationships in SZ.

## Limitations

Our study has several limitations. First, publication bias is almost inevitable, although we conducted a comprehensive literature search [121]. Second, the correlation between outcome-evoked activation and other clinical variables, such as the duration of illness, was not investigated because of insufficient data. Third, we cannot rule out the potential influence of medication, the stage of illness and several methodological factors on our results. Medication history or stages of disease varied in the included samples, which leads to the heterogeneity of brain activity during the reward anticipation phase. Future longitudinal studies are needed to investigate the effects of both medication and the stage of disease on neural dysfunctions in reward processing. In addition, the included articles used different MRI scanners with different MRI field strengths, which may lead to methodological heterogeneity and potentially limit our ability to detect robust group differences. Fourth, we included only studies on adult patients in our analysis, and the generalizability of our findings in children/adolescents needs to be further tested. Finally, since computational approaches are useful to reveal hidden psychological states subtending motivation and experienced pleasure, a systematic investigation of complex learning components may help to clarify these hidden states.

## Conclusion

The present study examined neural mechanisms underlying different phases of reward processing in SZ patients and their relevance to clinical symptomology. Patients with SZ showed hypoactivation in the mesocorticolimbic circuit during reward anticipation and elevated activation in the striatal-limbic circuitry but reduced responses in the DLPFC and mPFC were elicited by monetary outcomes. Anticipation-evoked VS hypoactivation was linked to negative symptoms, and outcome-evoked mPFC activation was linked to positive symptoms. Our findings showed dissociated neurobiological mechanisms in different aspects of reward processing and have the potential to clarify the complex brain-behavior relationships in SZ.

## Supplementary information

Supplementary Information

Supplementary Figure S1
