# Supplementary material for: Neural substrates of reward anticipation and outcome in schizophrenia: a meta-analysis of fMRI findings in the monetary incentive delay task
Source: Transl Psychiatry. 2022 Oct 16;12:448. doi: 10.1038/s41398-022-02201-8 (PMC9573872; doi:10.1038/s41398-022-02201-8)
Supplement: Supplementary file 1 — Supplementary Information [file 41398_2022_2201_MOESM1_ESM.docx]

**Neural substrates of reward anticipation and outcome in schizophrenia: a meta-analysis of fMRI findings in the monetary incentive delay task**

Jianguang Zeng, PhD^1△^, Jiangnan Yan, PhD ^1△^, Hengyi Cao, PhD^2,3△^, Yueyue Su, MS^4^,

Yuan Song, MS^4^, Ya Luo, MD^5^, Xun Yang, PhD^4※^

**Supplementary Information**

Description of monetary incentive delay task 1

Extended Methods for details on complementary analyses 3

Sensitivity analysis 3

Subgroup analyses 3

Regression analyses 3

Moderation analysis 4

Supplemental References 5

Figure legend 6

Table S1 Summary of the methods and results of brain activation in SZ in MID-related fMRI studies 7

Table S2 Subgroup and jackknife sensitivity analyses in brain activation difference between SZ and HC during reward anticipation stage 12

Table S3 Moderation analysis of the % SGA users on the negative symptom and striatum activity during reward anticipation 13

Table S4 Subgroup and jackknife sensitivity analyses of brain activation difference between SZ and HC during reward outcome stage 14

Table S5 Moderation analysis of the % SGA users on the positive symptom and mPFC activity during reward outcome 16

Description of monetary incentive delay task

The monetary incentive delay (MID) task, designed by Knutson and colleagues, aims to “localize” reward responses deep in the brain through exploiting the spatial and temporal resolution of functional magnetic resonance imaging [1]. Briefly, the MID task involves two stages: a) reward anticipation, where the visual stimuli (i.e. win; lose; neutral) are visualized before action; and b) reward outcome, where the trial outcome and total outcome are presented after action [2]. Importantly, this task, integrated within theories of human decision-making and reward processing, has been widely used and validated to probe psychiatric disorders (e.g., schizophrenia, depression, addiction) [3,4]. Previous studies suggested that the MID task is a robust method with excellent reliability and validity to delineate behavioral and biological processes underlying different reward stages, especially within the striatum [5,6].

The outline of the classical MID task experimental paradigm is shown in Figure S1. A cue (visual cue; 500 ms) that indicate the trial condition, win (up arrow), lose (down arrow) or neutral (arrow pointing left and right) is shown first. After a delay (mean delay: 4 000 ms), a visual target appeared on the screen, participants need to identify clues and press the button as soon as possible, so as to obtain a reward or avoid loss (target: 500 ms). The process of cue presentation is known as the anticipation stage. Then, participants received feedback about the trial outcome and total outcome (feedback: 500 ms) and a short delay (delay: 1 000 ms). This period refers to the outcome stage [2]. Duration of one trial was 6 seconds, and a mean intertrial interval (3 530 ms) was inserted between each trial. The modified MID task, adding or reducing the type of incentive cues, or introducing the probability information of each reward cue based on the classic MID task, has the same flow as the classic MID paradigm.

Several unique advantages of this paradigm are listed below: First, the MID task is relatively simple and only requires participants to respond to the target instead of making complex guesses or decisions. Second, the interaction between valence and temporal phase could be modeled in this task [4,7]. Third, it allows the detailed examination of different stages of reward processing. That is, the anticipation period can be distinguished from an outcome period by inserting a target reaction [8].

Extended Methods for details on [complementary analyses](#_Toc84496946)

Complementary analyses (jackknife sensitivity analyses, subgroup analyses, regression analyses and moderation analyses) were conducted in both the anticipation meta-analysis and the outcome meta-analysis.

Sensitivity analysis

The whole-brain jackknife sensitivity analysis aimed to assess the reproducibility of the results. This is mainly achieved by repeating the main statistical analysis for n-1 (n = the number of datasets included) times, but deleting one study each time. A brain region in the main meta-analysis remained significant in all or most of the study combinations means a highly robust result.

Subgroup analyses

Considering the possible heterogeneity caused by different clinical and imaging methodological variables, we also performed subgroup analyses in the anticipation meta-analysis and the outcome meta-analysis. The subgroup analyses were repeated several times, including only homogeneous studies each time. Specifically, we conducted subgroup analyses for those studies only including chronic SZ, for those including SZ patients diagnosed by DSM, for those including SZ patients receiving medication treatment, for those using a 3-T MRI scanner, for those using SPM software and for those reporting coordinates corrected for comparisons.

Regression analyses

Meta-regression analyses were conducted to test the effect of potential confounding variables and moderators on abnormal task-evoked activation. Several factors were considered: the mean age, the percentage of males, the duration of illness, the PANSS scores (PANSS-T; PANSS-P, PANSS-N), the % of SGA users and the % of FGA users, For the reward outcome, the duration of illness could not be explored due to a lack of sufficient data. The simple linear regression, weighted by the squared root of the sample size and restricted to predict only the possible SDM values, was used to investigate the potential effects of variables above [9]. Furthermore, we discarded findings outside those regions detected in the main analyses [9].

Moderation analysis

The moderation analysis tests whether the relationship between two variables is dependent on a third variable (W). The moderating effect is assessed with the regression equation [10]:

$$Y=a_{0}+a_{1}X+a_{2}W+a_{3}XW+r$$

Significant relation between the independent variable and the dependent variable is a precondition for moderation analysis. Additionally, the interaction of the predictor and the moderator (X × W) is required to significantly predict the dependent variable, namely $a_{3}$ (B) is significant at *p* < 0.05.

Supplemental References

1. Knutson B, Westdorp A, Kaiser E, Hommer D. FMRI visualization of brain activity during a monetary incentive delay task. *Neuroimage* 2000;12:20-27.

2. Wilson RP, Colizzi M, Bossong MG, Allen P, Kempton M, Mtac, et al. The Neural Substrate of Reward Anticipation in Health: A Meta-Analysis of fMRI Findings in the Monetary Incentive Delay Task. *Neuropsychol Rev* 2018;28:496-506.

3. Knutson B, Heinz A. Probing psychiatric symptoms with the monetary incentive delay task. *Biol Psychiatry* 2015;77:418-420.

4. Balodis IM, Potenza MN. Anticipatory reward processing in addicted populations: a focus on the monetary incentive delay task. *Biol Psychiatry* 2015;77:434-444.

5. Pegg S, Jeong HJ, Foti D, Kujawa A. Differentiating stages of reward responsiveness: Neurophysiological measures and associations with facets of the behavioral activation system. *Psychophysiology* 2021;58:e13764.

6. Volman I, Pringle A, Verhagen L, Browning M, Cowen PJ, Harmer CJ. Lithium modulates striatal reward anticipation and prediction error coding in healthy volunteers. *Neuropsychopharmacology* 2021;46:386-393.

7. Oldham S, Murawski C, Fornito A, Youssef G, Yucel M, Lorenzetti V. The anticipation and outcome phases of reward and loss processing: A neuroimaging meta-analysis of the monetary incentive delay task. *Hum Brain Mapp* 2018;39:3398-3418.

8. Rademacher L, Krach S, Kohls G, Irmak A, Grunder G, Spreckelmeyer KN. Dissociation of neural networks for anticipation and consumption of monetary and social rewards. *Neuroimage* 2010;49:3276-3285.

9. Radua J, Mataix-Cols D. Voxel-wise meta-analysis of grey matter changes in obsessive-compulsive disorder. *Br J Psychiatry* 2009;195:393-402.

10. Preacher KJ, Rucker DD, Hayes AF. Addressing Moderated Mediation Hypotheses: Theory, Methods, and Prescriptions. *Multivariate Behav Res* 2007;42:185-227.

Figure legend

**Figure S1** **Outline of the MID experimental paradigm**

The MID task consists of two parts, the anticipation phase of presenting reward cues (i.e., win: up arrow; neutral: arrow pointing left and right; lose: down arrow) and the outcome phase of receiving feedback.

Abbreviations: ITI = intertrial interval; ms = millisecond.

Table S1 Summary of the methods and results of brain activation in SZ in MID-related fMRI studies

| **Study** | **Research method** | **Task**  **phases** | **Brain regions with group differences** | | **Relationship between brain activity and symptoms** | |
| --- | --- | --- | --- | --- | --- | --- |
|  |  |  | **SZ < HC** | **SZ > HC** | **Negative symptoms** | **Positive symptoms** |
| Abler et al. 2008 | WB  +  ROI | Anticipation | ns. | — | — | — |
|  |  | Outcome | ns. | — | — | — |
| Alves et al. 2013 | WB  +  ROI | Anticipation | STG; PCC; Inferior frontal gyrus; Lentiform nucleus; Superior frontal gyrus; Cingulate gyrus; MFG; ACC; L VS* | — | — | — |
| Arrondo et al. 2015 | WB  +  ROI | Anticipation | R VS | — | activation of VS was negatively correlated with depression and anhedonia symptoms | — |
| Esslinger et al. 2012 | WB  +  ROI | Anticipation | R VS* | — | — | activation of VS was negatively correlated with delusions and hostility symptoms |
| Gilleen et al. 2015 | WB | Anticipation | ns. | — | — | — |
|  |  | Outcome | ns. | — | — | — |
| Hägele et al. 2012 | WB  +  ROI | Anticipation | R VS | — | activation of VS was negatively correlated with depression symptoms | — |
| Juckel et al. 2006a | WB  +  ROI | Anticipation | L VS* | — | activation of VS was negatively correlated with negative symptoms | — |
| Juckel et al. 2006b | ROI | Anticipation | VS* | — | activation of left VS was negatively correlated with negative symptoms | — |
| Kaliuzhna et al. 2020 | ROI | Anticipation | — | — | activation of left VS was negatively correlated with motivation | — |
| Kirschner et al. 2016a | ROI | Anticipation | ns. | — | — | activation of right VS was positively correlated with positive symptoms |
| Kirschner et al. 2016b | ROI | Anticipation | ns. | — | activation of VS was negatively correlated with apathy | — |
|  |  | Outcome | — | R VS* | — | — |
| Kirschner et al. 2016c | ROI | Outcome | — | Caudate*; Anterior insula/inferior frontal gyrus* | activation of right caudate was negatively correlated with negative symptoms | — |
| Kirschner et al. 2018 | ROI | Outcome | R caudate* | — | activation of the right striatum was negatively correlated with negative symptoms | — |
| Kirschner et al. 2020 | ROI | Anticipation | ns. | — | — | — |
| Kluge et al. 2018 | ROI | Anticipation | — | — | activation of VS was negatively correlated with negative symptoms | — |
| Koch et al. 2015 | WB | Anticipation | L MFG; Precentral gyrus; R MCC; R Parahippocampal gyrus; Caudate body; L Putamen; R Thalamus; R Amygdala; R STG; R ITG; L Fusiform gyrus; L Precuneus; R Cuneus; Middle occipital gyrus; R Brain stem; R Vermis; Cerebellum | — | activation of VS was negatively correlated with negative symptoms | — |
| Li et al. 2017 | WB  +  ROI | Anticipation | R Thalamus; Insula; Dorsal Caudate; DS; VS*; L Cingulate gyrus; R Subcallosal gyrus; R ACC; | — | — | — |
|  |  | Outcome | — | R Lateral globus pallidus; R Putamen; R Thalamus; L Globus pallidus; L Medial globus pallidus; VS; DS* | — | — |
| Mucci et al. 2015 | WB | Anticipation | ns. | — | activation of DS was negatively correlated with avolition | — |
|  |  | Outcome | ns. | — | — | — |
| Nielsen et al. 2012a | WB  +  ROI | Anticipation | STG; Precentral gyrus; Tuber; Postcentral gyrus; inferior occipital gyrus; Thalamus; Precuneus; Middle occipital gyrus; Caudate; Sub-gyral; Cingulate gyrus; PCC; Cuneus; ITG; Lingual gyrus; Inferior Parietal lobule; Transverse temporal gyrus; Sub-temporal gyrus; VS*; VTA* | — | — | activation of VS was negatively correlated with positive symptoms |
|  |  | Outcome | — | DLPFC* | — | — |
| Nielsen et al. 2012b | WB  +  ROI | Anticipation | VS* | — | — | activation of VS was positively correlated with positive symptoms |
| Nielsen et al. 2017 | WB  +  ROI | Anticipation | Basal ganglia | — | activation of left caudate was negatively correlated with negative symptoms | — |
| Schlagenhauf et al. 2008 | WB  +  ROI | Anticipation | R VS* | — | activation of left VS was negatively correlated with negative symptoms | — |
| Schlagenhauf et al. 2009 | WB  +  ROI | Anticipation | R VS | — | — | — |
|  |  | Outcome | mPFC; VS | — | — | activation of mPFC was negatively correlated with delusional symptom |
| Schwarz et al. 2019 | WB  +  ROI | Anticipation | R VS* | — | — | — |
|  |  | Outcome | — | R Fusiform; B Putamen; R Precentral; B ACC; Cerebellum; | — | — |
| Smucny et al. 2021 | ROI | Anticipation | dACC*; right insula*; VS* | — | — | — |
|  |  | Outcome | VmPFC*; subgenual ACC*; R VS* | — | — | — |
| Simon et al. 2010 | ROI | Anticipation | ns. | — | activation of VS was negatively correlated with apathy | — |
|  |  | Outcome | ns. | — | activation of VS was negatively correlated with depressive symptoms | — |
| Stepien et al.  2018 | WB  +  ROI | Anticipation | ns. | — | activations of VS and DS were negatively correlated with apathy | — |
| Subramaniam et al. 2015 | WB  +  ROI | Anticipation | VS*; L Occipital cortex; R MFG | — | — | activations of mPFC and MCC were positively correlated with positive symptoms |
|  |  | Outcome | L SFG; MFG; ACC; R MFG | — | — | — |
| Walter et al. 2009 | WB | Anticipation | Dorsal ACC | — | — | activation of ACC was negatively correlated with positive symptoms |
|  |  | Outcome | VPFC; Anterior insula | L VS | — | — |
| Waltz et al. 2010 | WB  +  ROI | Anticipation | — | — | activation of left VS was negatively correlated with anhedonia | — |
|  |  | Outcome | — | — | activation of mPFC was negatively correlated with avolition and anhedonia | activation of mPFC was negatively correlated with positive symptoms |

Note: WB = whole brain; ROI = regions of interest; ns. = no significant group difference; VS = ventral striatum; DS = dorsal striatum; STG = superior temporal gyrus; ACC = anterior cingulate cortex; PCC = posterior cingulate cortex; MCC = middle cingulate gyrus; mPFC = medial prefrontal cortex; VPFC = ventrolateral prefrontal cortex; VTA = ventral tegmental area; SFG = superior frontal gyrus; MFG = medial frontal gyrus; ITG = inferior temporal gyrus; R = right; L = left; * = based on ROI approach; — = the relevant results were not reported.

Table S2 Subgroup and jackknife sensitivity analyses in brain activation difference between SZ and HC during reward anticipation stage

| **Studies** | **Striatum** | **ACC & MCC** | **R** **precentral gyrus** | **R STG** |
| --- | --- | --- | --- | --- |
| **Subgroup analyses** | | | |  |
| Studies only including chronic SZ (n=14) | Y | Y | Y | N |
| Studies including SZ patients diagnosed by DSM (n=15) | Y | Y | Y | Y |
| Studies including SZ patients receiving medication treatment (n=13) | Y | Y | Y | N |
| Studies using a 3-T MRI scanner (n=13) | Y | Y | Y | N |
| Studies using SPM software (n=13) | Y | Y | Y | Y |
| Studies reporting coordinates corrected for comparisons (n=14) | Y | Y | N | Y |
| **Sensitivity analyses** | | | |  |
| Abler et al. 2008 | Y | Y | Y | Y |
| Alves et al. 2013 | Y | Y | Y | Y |
| Arrondo et al. 2015 | Y | Y | Y | Y |
| Esslinger et al. 2012 | Y | Y | Y | Y |
| Gilleen et al. 2015 | Y | Y | Y | Y |
| Juckel et al. 2006 | Y | Y | Y | Y |
| Koch et al. 2015 | Y | Y | Y | N |
| Li et al. 2017 | Y | Y | Y | Y |
| Mucci et al. 2015 | Y | Y | Y | Y |
| Nielsen et al. 2012 | Y | Y | Y | Y |
| Schlagenhauf et al. 2008 | N | N | N | N |
| Schlagenhauf et al. 2009 | Y | Y | Y | Y |
| Schwarz et al. 2019 | Y | Y | Y | Y |
| Stepien et al. 2018 | Y | Y | Y | Y |
| Subramaniam et al. 2015 | Y | Y | N | Y |
| Walter et al. 2009 | Y | Y | Y | Y |
| Waltz et al. 2010 | Y | Y | Y | Y |

Note: DSM = diagnostic and statistical manual of mental disorders; ACC = anterior cingulate cortex; MCC = median cingulate cortex; STG = superior temporal gyrus; L = left; R = right; Y = yes; N = no.

Table S3 Moderation analysis of the % of SGA users on the negative symptom and striatum activity during reward anticipation

| **Variables** | **BOLD (right striatum)** | | | | | | |
| --- | --- | --- | --- | --- | --- | --- | --- |
|  | **Model 1** | | |  | **Model 2** | | |
|  | ***B*** | ***t*** | ***p*** |  | ***B*** | ***t*** | ***p*** |
| Negative symptoms | -0.039 | -1.281 | 0.221 |  | -0.176 | -3.081 | 0.009 |
| The % of SGA users | -0.041 | -0.132 | 0.897 |  | -3.837 | -2.661 | 0.020 |
| Symptoms × SGA |  |  |  |  | 0.173 | 2.676 | 0.019 |
| R^2^ | 0.258 | | |  | 0.522 | | |

Note: % of SGA users = the proportion of schizophrenia who had ever received second-generation antipsychotics.

Table S4 Subgroup and jackknife sensitivity analyses of brain activation difference between SZ and HC during reward outcome stage

| **Studies** | **Activation** | | | | |  |  | **Deactivation** | | |
| --- | --- | --- | --- | --- | --- | --- | --- | --- | --- | --- |
|  | **R**  **striatum** | **L**  **striatum** | **L**  **cerebellum** | **R**  **parahippocampal gyrus** | **R**  **postcentral gyrus** | **R**  **MCC** |  | **mPFC** | **L**  **DLPFC** | **R**  **DLPFC** |
| **Subgroup analyses** | | | |  |  |  |  |  |  |  |
| Studies only including chronic SZ (n=9) | Y | Y | Y | Y | Y | Y |  | Y | Y | Y |
| Studies including SZ patients diagnosed by DSM (n=8) | Y | Y | Y | Y | Y | Y |  | Y | Y | Y |
| Studies including SZ patients who receiving medication treatment (n=8) | Y | Y | Y | Y | Y | Y |  | Y | Y | Y |
| Studies using a 3-T MRI scanner (n=9) | Y | Y | Y | Y | Y | Y |  | Y | Y | Y |
| Studies using SPM software (n=8) | Y | Y | Y | Y | Y | Y |  | Y | Y | Y |
| Studies reporting coordinates corrected for comparisons (n=7) | Y | Y | Y | Y | Y | Y |  | Y | Y | Y |
| **Sensitivity analyses** | | | |  |  |  |  |  |  |  |
| Abler et al. 2008 | Y | Y | Y | Y | Y | Y |  | Y | Y | Y |
| Gilleen et al. 2015 | Y | Y | Y | Y | Y | Y |  | Y | Y | Y |
| Li et al. 2017 | N | Y | Y | Y | Y | Y |  | Y | Y | Y |
| Mucci et al. 2015 | Y | Y | Y | Y | Y | Y |  | Y | Y | Y |
| Nielsen et al. 2012 | Y | Y | Y | Y | Y | Y |  | Y | Y | Y |
| Schwarz et al. 2019 | Y | Y | N | Y | Y | N |  | Y | Y | Y |
| Subramaniam et al. 2015 | Y | Y | Y | Y | Y | Y |  | N | N | N |
| Walter et al. 2009 | Y | Y | Y | Y | Y | Y |  | Y | Y | Y |
| Schlagenhauf et al. 2009 | Y | Y | Y | Y | Y | Y |  | Y | Y | Y |
| Waltz et al. 2010 | Y | Y | Y | Y | Y | Y |  | Y | Y | Y |

Note: DSM = diagnostic and statistical manual of mental disorders; MCC = median cingulate cortex; mPFC = medial prefrontal cortex; DLPFC = dorsolateral prefrontal cortex; L = left; R = right; Y = yes; N = no.

Table S5 Moderation analysis of the % of SGA users on the positive symptom and mPFC activity during reward outcome

| **Variables** | **BOLD (mPFC)** | | | | | | |
| --- | --- | --- | --- | --- | --- | --- | --- |
|  | **Model 1** | | |  | **Model 2** | | |
|  | ***B*** | ***t*** | ***p*** |  | ***B*** | ***t*** | ***p*** |
| Positive symptoms | 0.360 | -.997 | .357 |  | -.229 | -7.521 | .001 |
| The % of SGA users | -0.041 | .618 | .559 |  | -4.816 | -6.251 | .002 |
| Symptoms × SGAs |  |  |  |  | 0.230 | 6.945 | .001 |
| R^2^ | 0.496 | | |  | 0.953 | | |

Note: % of SGA users = the proportion of schizophrenia who had ever received second-generation antipsychotics.
