# Supplementary figures and images for: Neural substrates of reward anticipation and outcome in schizophrenia: a meta-analysis of fMRI findings in the monetary incentive delay task
Source: Transl Psychiatry. 2022 Oct 16;12:448. doi: 10.1038/s41398-022-02201-8 (PMC9573872; doi:10.1038/s41398-022-02201-8)

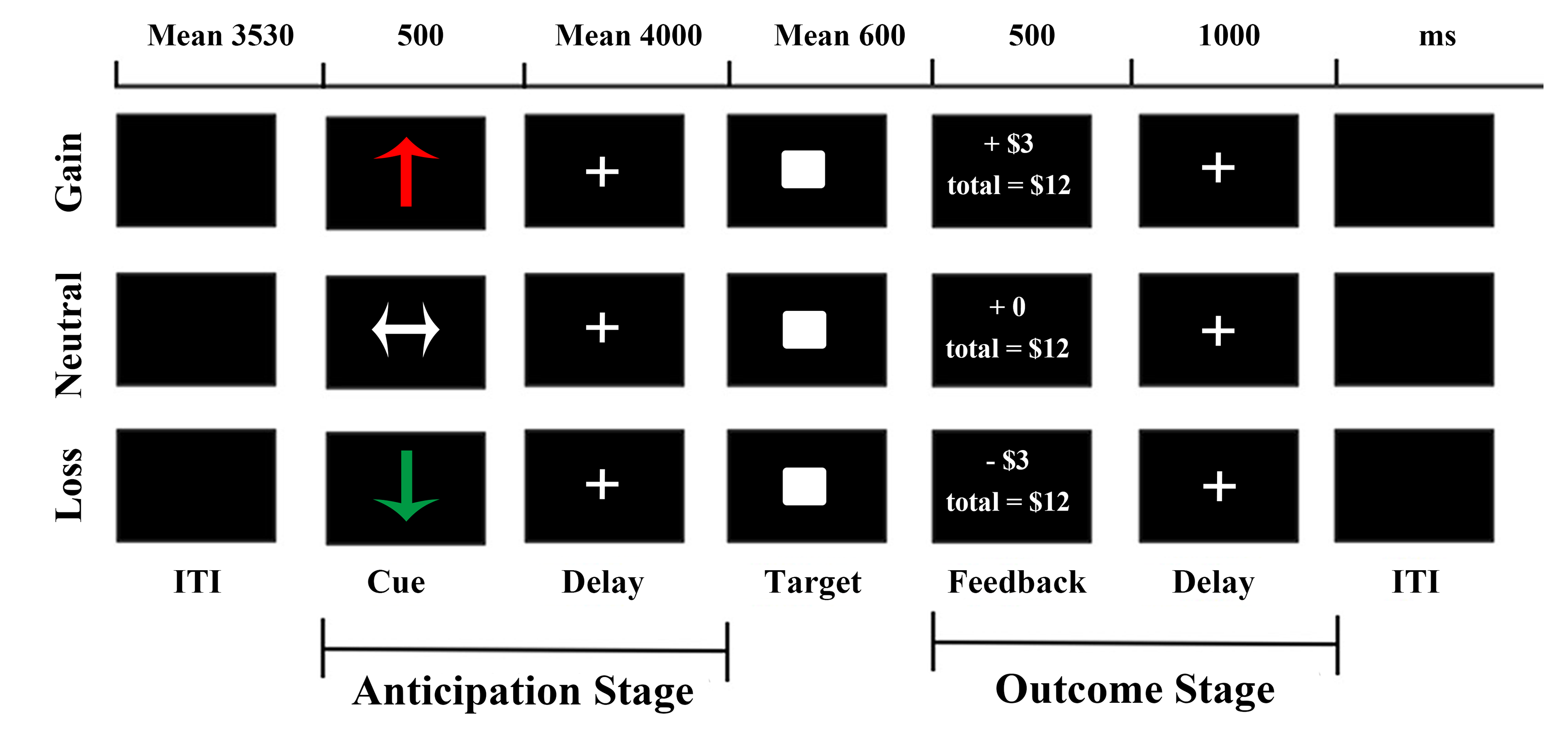

Supplement: Supplementary file 2 — Supplementary Figure S1 [file 41398_2022_2201_MOESM2_ESM.tif]
